# A Patient with Proopiomelanocortin Deficiency: An Increasingly Important Diagnosis to Make

**DOI:** 10.4274/jcrpe.4638

**Published:** 2018-02-26

**Authors:** Semra Çetinkaya, Tülay Güran, Erdal Kurnaz, Melikşah Keskin, Elif Sağsak, Senay Savaş Erdeve, Jenifer P. Suntharalingham, Federica Buonocore, John C. Achermann, Zehra Aycan

**Affiliations:** 1 University of Health Sciences, Dr. Sami Ulus Training and Research Hospital, Clinic of Children’s Health and Disease, Health Implementation and Research Center, Ankara, Turkey; 2 Marmara University Faculty of Medicine, Department of Pediatric Endocrinology, İstanbul, Turkey; 3 University College London, UCL Great Ormond Street Institute of Child Health, London, United Kingdom; 4 Yıldırım Beyazıt University Faculty of Medicine, Department of Pediatric Endocrinology, Ankara, Turkey

**Keywords:** Obesity, melanocortin 4 receptors, paediatric obesity, proopiomelanocortin deficiency

## Abstract

Proopiomelanocortin (POMC) deficiency is a rare monogenic disorder with early-onset obesity. Investigation of this entity have increased our insight into the important role of the leptin-melanocortin pathway in energy balance. Here, we present a patient with POMC deficiency due to a homozygous c.206delC mutation in the POMC gene. We discuss the pathogenesis of this condition with emphasis on the crosstalk between hypothalamic and peripheral signals in the development of obesity and the POMC-melanocortin 4 receptors system as a target for therapeutic intervention.

## What is already known on this topic?

Proopiomelanocortin (POMC) deficiency is an extremely rare disorder characterized by early-onset obesity, adrenal insufficiency, red hair and decreased skin pigmentation. Hyperphagia, cholestasis, exponential weight gain and adrenal insufficiency are typically observed during the first months of life. In some children, the diagnosis may only be established later.

## 

### What this study adds?

This study presents clinical and molecular features of a child with POMC deficiency. We also provide a brief summary of the clinical and genetic features of POMC deficiency based on previously published patient reports and describe how these are providing insight into the role of POMC in the regulation of human metabolism.

## Introduction

Proopiomelanocortin (POMC) is a 241-amino acid polypeptide that is cleaved via prohormone convertase (PC) to produce the peptides γ-, β-, α-melanocyte stimulating hormone (MSH), adrenocorticotropin hormone (ACTH), γ-, β-lipotrophin and endorphins (1). These peptides stimulate five different melanocortin receptors (MCR) with varying affinity and specificity. Cortisol secretion is regulated through MC2R in the adrenal gland while MC1R regulates skin pigmentation. MC3R and MC4R regulate body weight.

Congenital POMC deficiency develops due to genetic defects in the *POMC* gene located at *Chr.*2p23.3. This disorder is characterized by early-onset obesity, adrenal insufficiency, red hair and decreased skin pigmentation (1,2,3,4). Obesity develops as a result of inadequate production of α- and β-MSH, which normally activate the MC3R in the arcuate nucleus and the MC4R in the paraventricular nucleus and antagonize the action of agouti-related peptide (AgRP) (4). The hypocortisolaemia and hypopigmentation are due to inadequate stimulation of MC2R and MC1R by POMC-derived peptides in the adrenal gland and skin, respectively. POMC deficiency is rare, but has increased our insight into the important role of the leptin-melanocortin pathway in energy balance.

Here, we present the clinical characteristics of a patient with POMC deficiency due to a mutation in the POMC gene, hoping to contribute to a better understanding of the leptin-melanocortin pathway and to introduce possible treatment options.

## Case Report

**Figure 1:**
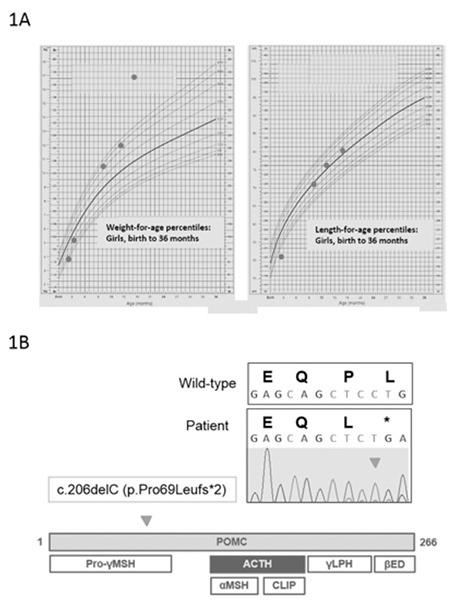
Weight (left) and height chart (right) of the patient showing rapid weight gain after infancy (A). Chromatogram showing the homozygous c.206delC change that results in a leucine residue (CTG) being replaced by a stop codon (TGA) (upper). This mutation causes disruption of proopiomelanocortin and prevents cleavage of proopiomelanocortin into key peptides such as adrenocorticotropin hormone and α-melanocyte stimulating hormone (lower) (B) ACTH: adrenocorticotropin hormone, POMC: proopiomelanocortin, CLIP: corticotropin-like intermediate lobe peptide, γLPH: γ-lipotropin, βED: β-endorphin, MSH: melanocyte-stimulating hormone

This female patient presented at age 2.5 months with restlessness, cyanosis, and spasms. She was found to be hypoglycaemic with a blood glucose of 31 mg/dL. She was born at 39 weeks gestation with a birth weight of 3000 grams and had no problems during the prenatal or early postnatal period. Her mother and father were not related and she had a healthy brother aged five years. There was no history of relevant disease in the family. Examination findings at presentation revealed growth failure (body weight: 3700 g, 3^rd^ percentile; height: 51 cm, <3^rd^ percentile; head circumference: 35 cm, <3^rd^ percentile) (Figure 1A), red eyebrows and hair and normal female genitalia. Results of further laboratory investigations confirmed the hypoglycaemia (blood glucose: 19 mg/dL) and revealed mild hyponatraemia with a sodium of 132 mmol/L (135-143), accompanied by a potassium of 4.8 mmol/L (3.1-5.5), mildly elevated aspartate transaminase: 123 U/L (<36) and creatinine kinase: 419 U/L (34-204).

Concomitant with the hypoglycaemic state (34 mgl/dL), serum and urinary ketones were low and there was no evidence of hyperinsulinaemia or any other metabolic cause for the hypoglycemia (serum insulin: 0.11 U/L, C-peptide: <0.03 nmol/L, lactate: 2.37 mmol/L (0.49-2.19), ammonium: 93.5 mmol/L (13.5-42.8), urinary and blood amino acids and organic acid profile normal). Total/indirect bilirubin levels were 3.7/2.4 mg/dL. However, the child was hypocortisolaemic (cortisol: <5.51 nmol/L) with an undetectable ACTH level (ACTH: <1.1 pmol/L). Other anterior pituitary hormones were as follows: growth hormone 14.8 mg/L; thyroid-stimulating hormone 1.73 U/L; free thyroxine 14.02 pmol/L; prolactin 390 mIU/L (3-24); follicle-stimulating hormone <3 U/L (0.1-3.3); luteinizing hormone <0.07 U/L (0-1.9). A low-dose ACTH stimulation test showed an insufficient cortisol response at 40 minutes (12.1 nmol/L). A magnetic resonance imaging (MRI) scan of the pituitary gland was normal. A diagnosis of isolated central (secondary) ACTH insufficiency, rather than panhypopituitarism was made.

Hydrocortisone treatment was initiated which subsequently enabled successful control of the hypoglycemia. Due to the presence of central adrenal insufficiency together with red hair, a genetic analysis of the *POMC* gene was undertaken. A homozygous frameshift mutation, c.206delC (p.P69Lfs*2) in the *POMC* gene was detected (5). This mutation results in a downstream frameshift and premature protein truncation, removing ACTH and other important peptides and most likely completely disrupting POMC function (Figure 1B).

**Figure 2:**
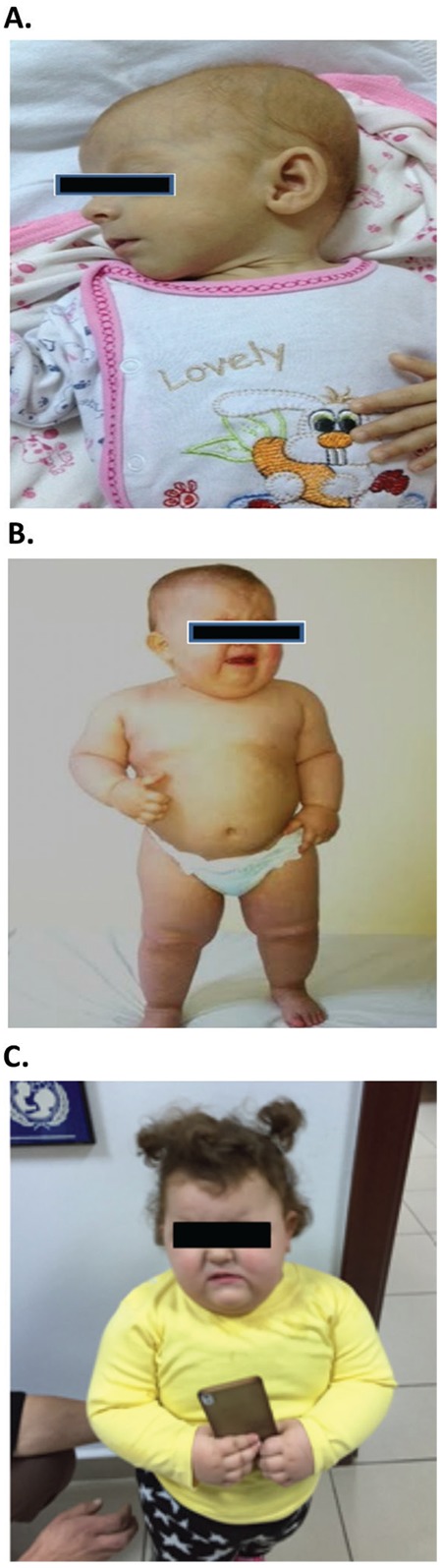
General appearance [At appointment (red eyebrows and hair) (A), at 1.5 years old (obesity) (B), at 2.5 years old (C)]

Following this initial presentation, in subsequent months, the child showed rapid increase in growth and developed obesity. At 17 months of age her weight was 16.9 kg (>97^th^ percentile), height 80 cm (50^th^ percentile), and head circumference 40 cm (75-90^th ^percentile) (Figure 1A). Her eyebrows and hair were red (Figure 2). The final steroid treatment dose was 8 mg/m^2^/day. An informed consent form for publication was given by the parents.

## Discussion

Congenital isolated ACTH deficiency is a rare condition and the symptoms and signs can be nonspecific. However, it can be life threatening unless appropriate steroid replacement is initiated. POMC is synthesized in the corticotropic cells of the pituitary gland by the action of the transcription factor TBX19/TPIT. POMC is then cleaved to form ACTH by the enzyme PC1 [PC1/3, proprotein convertase subtilisin/kexin (PCSK) type 1] following corticotropin-releasing hormone stimulation (Figure 1B) (6). Isolated ACTH deficiency can result from pathogenic variations of the *TBX19 *(TPIT), *PCSK1 *(PC1/3) and *POMC* genes (6).

**Figure 3:**
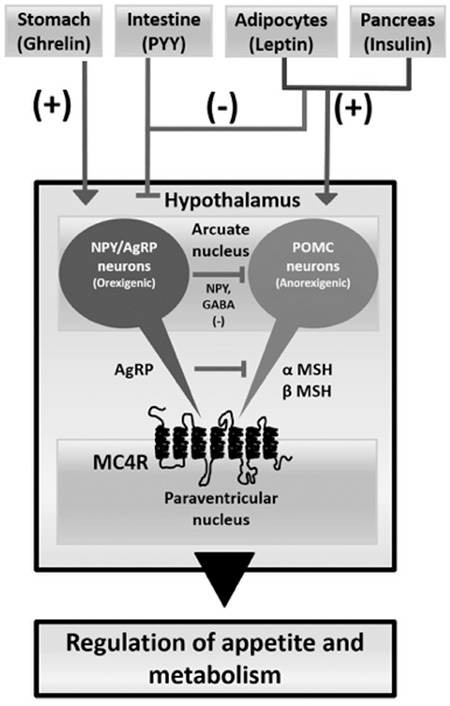
Cartoon showing potential interactions of proopiomelanocortin neurons in appetite regulation. In the hypothalamus, nutrition and energy hemostasis is balanced by proopiomelanocortin and agouti-related peptide/neuropeptide Y through melanocortin receptors. This system is regulated by peripheral polypeptides such as leptin, ghrelin, insulin and peptide YY. In proopiomelanocortin deficiency, the appetite-stimulating effect of agouti-related peptide is not balanced by the appetite-suppressing effect of proopiomelanocortin. AgRP is co-expressed with neuropeptide Y. This peptide increases appetite and decreases energy use and metabolism. This system is mainly inhibited by leptin and stimulated by ghrelin NPY: neuropeptide Y, AgRP: agouti-related peptide, POMC: proopiomelanocortin, MSH: melanocyte-stimulating hormone, MC4R: melanocortin 4 receptors, PPY: peptide YY, GABA: gammα-amino butyric acid

Although POMC defects were first reported in 1998, relatively few children with the condition have been reported to date (4). The classic triad of POMC deficiency consists of early-onset obesity, central adrenal insufficiency and red hair. Hyperphagia (80-99%), cholestasis (30-79%, at onset), exponential weight gain (100%) and adrenal insufficiency (30-79%, at onset) are typically observed during the first months of life, but the diagnosis may only be established later in some children. Linear growth is initially normal, as in our patient, and weight gain may not occur initially in a child with uncontrolled adrenal insufficiency. However, weight often increases to above the 90^th^ percentile by the end of the first year. This process likely reflects an insufficiency of hypothalamic POMC. Normally, nutrition and energy hemostasis is balanced by the complex interaction of POMC and AgRP/neuropeptide Y (NPY) with MCR in the hypothalamus (Figure 3) (1,2,3,4). This system is also regulated by peripheral polypeptides such as leptin and ghrelin. Our patient showed a rapid and early-onset weight gain as a result of this process. The weight gain was independent of steroid treatment as only a physiological replacement dose of hydrocortisone was used and subsequent linear growth rate was stabilized on the 50^th^ percentile line, despite ongoing rapid weight gain.

The red hair associated with POMC deficiency is an important sign, especially in children from an ancestral background of dark hair. However, there are a few reports of children with POMC deficiency who do not have red hair or where only the roots of the hair are red (6,7,8,9). Other children may have red hair initially but this turns brown in the first three to four years of life (10).

Other reported features potentially associated with POMC deficiency include pale skin (Fitzpatrick type 1) due to reduced stimulation of MC1R by MSH; central hypothyroidism, possibly due to interactions between POMC and thyrotropin-releasing hormone in the hypothalamus (10,11,12,13); and hypogonadotropic hypogonadism with pubertal growth hormone deficiency reflecting a possible direct interaction between POMC and gonadotropin-releasing hormone neurons or indirectly via kisspeptin and NPY/AgRP. Our patient did not show hypothyroidism, but did have pale skin and had a low gonadotropin level in early infancy at around the time of the typical “minipuberty”. This finding may reflect impaired gonadotropin release so it is important to monitor the development of puberty in these patients. Transient hyponatraemia has been reported with central ACTH insufficiency, as was seen in our patient (14). This may reflect decreased free water clearance due to hypocortisolaemia, especially with intercurrent infections, or a supportive mineralocorticoid effect of cortisol at times of stress. Detecting hyponatraemia can sometimes lead to a misdiagnosis of primary adrenal insufficiency instead of a secondary or central defect. Finally, developmental delay with abnormal MRI changes has been reported in one child with POMC deficiency. This finding is likely to represent the effects of recurrent hypoglycaemia rather than the underlying condition itself (15).

Genetic analysis was useful to establish the diagnosis of POMC deficiency. Using a custom adrenal array coupled with next generation sequencing we identified a homozygous c.206delC mutation in the child (5). This nucleotide deletion was confirmed by Sanger sequencing and causes a frameshift and premature truncation of the protein at codon 70 (p.Pro69Leufs2*) (Figure 1B). This mutation results in a POMC product that lacks ACTH, α-MSH and other small peptides, and may be subject to non-sense mediated decay. A review of the literature shows that all reported patients with POMC deficiency have homozygous or compound heterozygous mutations in the amino-terminal region of the protein that result in defective ACTH and α-MSH synthesis. The c.206delC change has been reported in two other families in Turkey suggesting a founder effect (7). The only established point mutation in POMC causing a similar phenotype is p.Arg145Cys change that corresponds to a p.Arg8Cys mutation in the ACTH peptide (16). This point mutation results in a bioinactive form of POMC/ACTH with a clinical phenotype of red hair, obesity and central adrenal insufficiency but with elevated ACTH levels on biochemical testing. In addition, isolated obesity has been reported in carriers of POMC mutations or in association with heterozygous point mutations in POMC (especially p.Arg236Gly) (7,17,18,19). Overview of the clinical and molecular features of patients with POMC insufficiency published to date were presented in Table 1 (Table 1). However, the parents of our child had BMIs of 23 kg/m^2^ and 27 kg/m^2^.

Treatment of POMC deficiency can be challenging. Patients require life-long glucocorticoid treatment using replacement doses. Mineralocorticoid replacement is not required. Hypothyroidism should be monitored and treated if present. Early onset obesity can be very difficult to treat beyond standard dietary and lifestyle measures, but the hyperphagic component is especially challenging. Krude et al (10) attempted intranasal ACTH treatment in two index cases with the ACTH peptide fragment identical to α-MSH. However, ACTH treatment at low doses during the first six weeks followed by a high dose (5 mg/day) did not produce a significant response in weight loss. Recently, setmelanotide (Rhythm Pharmaceuticals, Boston, Massachusetts, USA) has been developed as a novel MC4R agonist for the treatment of rare genetic disorders of obesity associated with defects in the MC4 pathway. This novel therapy is currently in Phase II trials in patients with POMC deficiency.

In summary, we present a Turkish child with POMC deficiency due to a potential founder POMC mutation. Routine genetic analysis in patients suspected of POMC deficiency is recommended not only to guide lonγ-term prognosis and tailor the personalized management of these patients *per se*, but also to enable discovery of breakthrough treatments for important public health problems such as obesity.

## Figures and Tables

**Table 1:**
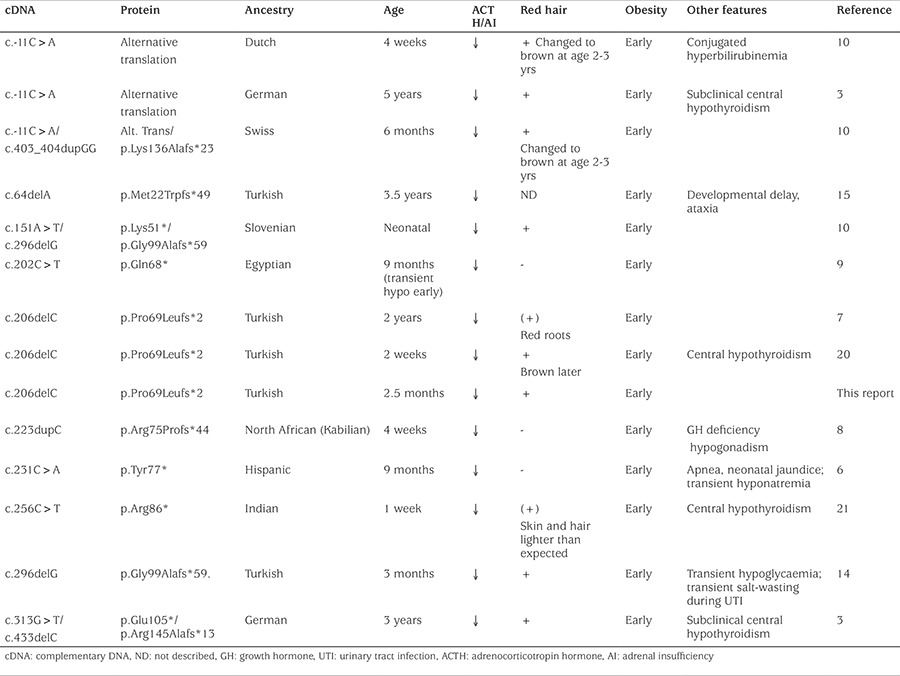
The clinical and molecular features of patients with POMC insufficiency reported to date

## References

[1] Biebermann H, Kühnen P, Kleinau G, Krude H (2012). The neuroendocrine circuitry controlled by POMC, MSH, and AGRP. Handb Exp Pharmacol.

[2] Krude H, Grüters A (2000). Implications of proopiomelanocortin (POMC) mutations in humans: the POMC deficiency syndrome. Trends Endocrinol Metab.

[3] Krude H, Biebermann H, Luck W, Horn R, Brabant G, Grüters A (1998). Severe early-onset obesity, adrenal insufficiency and red hair pigmentation caused by POMC mutations in humans. Nat Genet.

[4] Challis BG, Millington GWM, Pagon RA, Adam MP, Ardinger HH, Wallace SE, Amemiya A, Bean LJH, Bird TD, Fong CT, Smith RJH, Stephens K (1993-2015). Gene Reviews. Proopiomelanocortin Deficiency. GeneReviews® [Internet].

[5] Guran T, Buonocore F, Saka N, Ozbek MN, Aycan Z, Bereket A, Bas F, Darcan S, Bideci A, Guven A, Demir K, Akinci A, Buyukinan M, Aydin BK, Turan S, Agladioglu SY, Atay Z, Abali ZY, Tarim O, Catli G, Yuksel B, Akcay T, Yildiz M, Ozen S, Doger E, Demirbilek H, Ucar A, Isik E, Ozhan B, Bolu S, Ozgen IT, Suntharalingham JP, Achermann JC (2016). Rare causes of primary adrenal insufficiency: Genetic and clinical characterization of a large nationwide cohort. J Clin Endocrinol Metab.

[6] Mendiratta MS, Yang Y, Balazs AE, Willis AS, Eng CM, Karaviti LP, Potocki L (2011). Early onset obesity and adrenal insufficiency associated with a homozygous POMC mutation. Int J Pediatr Endocrinol.

[7] Farooqi IS, Drop S, Keogh JM, Biernacka J, Lowenbein S, Challis BG, O’Rahilly S (2006). Heterozygosity for a POMC-null mutation and increased obesity risk in humans. Diabetes.

[8] Clément K, Dubern B, Mencarelli M, Czernichow P, Ito S, Wakamatsu K, Barsh GS, Vaisse C, Leger J (2008). Unexpected endocrine features and normal pigmentation in a young adult patient carrying a novel homozygous mutation in the POMC gene. J Clin Endocrinol Metab.

[9] Cirillo G, Marini R, Ito S, Wakamatsu K, Scianguetta S, Bizzarri C, Romano A, Grandone A, Perrone L, Cappa M, Miraglia Del Giudice E (2012). Lack of red hair phenotype in a North-African obese child homozygous for a novel POMC null mutation: non sense-mediated decay RNA evaluation and hair pigment chemical analysis. Br J Dermatol.

[10] Krude H, Biebermann H, Schnabel D, Tansek MZ, Theunissen P, Mullis PE, Grüters A (2003). Obesity due to proopiomelanocortin deficiency: three new cases and treatment trials with thyroid hormone and ACTH4-10. J Clin Endocrinol Metab.

[11] Kim MS, Small CJ, Stanley SA, Morgan DG, Seal LJ, Kong WM, Edwards CM, Abusnana S, Sunter D, Ghatei MA, Bloom SR (2000). The central melanocortin system affects the hypothalamo-pituitarythyroid axis and may mediate the effect of leptin. J Clin Invest.

[12] Harris M, Aschkenasi C, Elias CF, Chandrankunnel A, Nillni EA, Bjøorbaek C, Elmquist JK, Flier JS, Hollenberg AN (2001). Transcriptional regulation of the thyrotropin-releasing hormone gene by leptin and melanocortin signaling. J ClinInvest.

[13] Amin A, Dhillo WS, Murphy KG (2011). The central effects of thyroid hormones on appetite. J Thyroid Res.

[14] Darcan S, Can S, Goksen D, Asar G (2010). Transient salt wasting in POMC-deficiency due to infection induced stress. Exp Clin Endocrinol Diabetes.

[15] Özen S, Özcan N, Uçar SK, Gökşen D, Darcan Ş (2015). Unexpected clinical features in a female patient with proopiomelanocortin (POMC) deficiency. J Pediatr Endocrinol Metab.

[16] Samuels ME, Gallo-Payet N, Pinard S, Hasselmann C, Magne F, Patry L, Chouinard L, Schwartzentruber J, René P, Sawyer N, Bouvier M, Djemli A, Delvin E, Huot C, Eugene D, Deal CL, Van Vliet G, Majewski J, Deladoëy J, FORGE Canada Consortium (2013). Bioinactive ACTH causing glucocorticoid deficiency. J Clin Endocrinol Metab.

[17] Challis BG, Pritchard LE, Creemers JW, Delplanque J, Keogh JM, Luan J, Wareham NJ, Yeo GS, Bhattacharyya S, Froguel P, White A, Farooqi IS, O’Rahilly S (2002). A missense mutation disrupting a dibasic prohormone processing site in pro-opiomelanocortin (POMC) increases susceptibility to early-onset obesity through a novel molecular mechanism. Hum Mol Genet.

[18] Buono P, Pasanisi F, Nardelli C, Ieno L, Capone S, Liguori R, Finelli C, Oriani G, Contaldo F, Sacchetti L (2005). Six novel mutations in the proopiomelanocortin and melanocortin receptor 4 genes in severely obese adults living in southern Italy. Clin Chem.

[19] Hasnain S (2015.). Prevalence of POMC R236G mutation in Pakistan. Obes Res Clin Pract.

[20] Ozen S, Aldemir O (2012). Early-onset severe obesity with ACTH deficiency and red hair in a boy: the POMC deficiency. Genet Couns.

[21] Hung CN, Poon WT, Lee CY, Law CY, Chan AY (2012). A case of early-onset obesity, hypocortisolism, and skin pigmentation problem due to a novel homozygous mutation in the proopiomelanocortin (POMC) gene in an Indian boy. J Pediatr Endocrinol Metab.

